# Association of drainage volume decline trajectories during the first 7 postoperative days with persistent seroma on postoperative day 30 after modified radical mastectomy for breast cancer

**DOI:** 10.3389/fonc.2026.1981532

**Published:** 2026-10-28

**Authors:** Qingzhao Bian, Xiangyu Wu, Mingkun Cui

**Affiliations:** Department of Thyroid and Breast Surgery I, Cangzhou Central Hospital, Cangzhou, Hebei, China

**Keywords:** breast cancer, drainage trajectory, modified radical mastectomy, persistent seroma, risk stratification

## Abstract

**Background:**

Persistent seroma after modified radical mastectomy (MRM) with axillary lymph node dissection (ALND) can prolong drainage and recovery. We identified drainage trajectories during the first postoperative week and evaluated their association with persistent seroma on postoperative day 30.

**Methods:**

This single-center retrospective study included 306 adults with unilateral invasive breast cancer who underwent MRM with level I–II ALND between 2021 and 2024. Trajectories of daily combined drainage volume were identified using latent class mixed-effects models. Firth penalized-likelihood logistic regression was used to adjust for age, body mass index, diabetes mellitus, neoadjuvant systemic therapy, resected specimen weight, operative time, and the number of dissected lymph nodes. Sensitivity analyses addressed uncertainty in class assignment, outcome definition, retention of the original drain, pathologic T category, and operating surgeon. Exploratory complete-case models compared trajectory classification with simpler drainage measures.

**Results:**

Rapid-decline (123 patients), moderate-decline (127 patients), and persistently high-output (56 patients) trajectories were identified. Persistent seroma occurred in 7.3%, 19.7%, and 57.1% of patients, respectively (*P* < 0.001). Relative to the rapid-decline trajectory, the adjusted odds ratios were 2.58 [95% confidence interval (CI), 1.10–6.07] and 11.44 (95% CI, 3.92–33.37), respectively. The association for the persistently high-output trajectory remained strong across sensitivity analyses, whereas that for the moderate-decline trajectory was less stable. The trajectory and postoperative day 7 (D7) drainage models showed similar discrimination. Adding trajectory class provided little incremental information beyond D7 drainage but improved model fit and discrimination beyond 7-day cumulative drainage and the relative decline from postoperative day 1 (D1) to D7.

**Conclusions:**

Persistently high output was consistently associated with persistent seroma on postoperative day 30. Trajectory classification should remain a research-oriented postoperative risk-stratification variable. The incremental clinical value of trajectory classification over the simpler D7 drainage measurement remains uncertain and requires prospective validation. Clinical application requires multicenter external validation, assessment of class-assignment reproducibility, calibration, clinically meaningful thresholds, and evaluation of clinical utility.

## 1 Introduction

Breast cancer remains a major contributor to the global burden of malignancy among women, and population growth and aging are expected to continue increasing the numbers of incident cases and deaths (1). Disease burden and attributable risk factors vary substantially among regions with different levels of sociodemographic development; perioperative care therefore remains an important modifiable component of the care pathway (2). For patients requiring total mastectomy and axillary management, the extent of axillary surgery should be selected according to nodal burden, response to preoperative treatment, and the need for locoregional control (3).

Mastectomy and axillary lymph node dissection create a large potential space, and seroma remains a common postoperative problem. The number of drains, criteria for drain removal, and approaches to fluid-collection management vary among institutions (4). Seroma formation involves lymphatic and microvascular injury, local inflammation, tissue exudation, and persistence of dead space; dead-space reduction and flap fixation can reduce the burden of fluid accumulation in some patients (5). Persistent seroma may necessitate repeated aspiration, drain reinsertion, or other treatment and may increase the burdens of infection and repeat intervention (6).

Closed-suction drainage facilitates early removal of exudate and promotes flap apposition; however, drain-related pain, restricted mobility, and the burden of home care can adversely affect the recovery experience (7). Changes in immune cells and inflammatory mediators within seroma fluid suggest that persistent fluid accumulation has a dynamic biological basis (8). A higher body mass index (BMI), larger breast volume, and more extensive resection have been associated with an increased risk of seroma (9).

Previous studies have generally relied on drainage volume on a single day, cumulative drainage volume, or time to drain removal, whereas longitudinal trajectories can simultaneously characterize drainage magnitude and rate of decline. Recent reviews and guidance have also emphasized the importance of standardizing seroma outcome definitions, drainage strategies, and reporting of interventions (10). This study identified drainage-volume decline trajectories during the first 7 postoperative days after modified radical mastectomy with level I–II axillary lymph node dissection and evaluated their associations with persistent seroma on postoperative day 30.

## 2 Materials and methods

### 2.1 Study population

This was a single-center retrospective study. Data were consecutively collected from patients who underwent modified radical mastectomy for breast cancer at our hospital between January 1, 2021, and December 31, 2024; the postoperative day-30 assessment of the last patient was completed on February 2, 2025. The inclusion criteria were as follows: age ≥18 years; unilateral primary invasive breast cancer confirmed by preoperative biopsy and postoperative pathology, with diagnosis and treatment based on clinical practice guidelines for early breast cancer (11); modified radical mastectomy preserving the pectoralis major muscle with level I–II axillary lymph node dissection and no simultaneous breast reconstruction, performed in accordance with contemporaneous breast cancer treatment standards (12); and complete drainage records for postoperative days 1–7 and complete 30-day outcome data. The exclusion criteria were as follows: bilateral breast surgery or another major concomitant operation; previous ipsilateral breast or axillary surgery or radiotherapy; distant metastasis before surgery; reoperation within 7 postoperative days because of bleeding or another reason; drain obstruction, accidental dislodgement, or abnormal drainage records; and missing key exposure or 30-day outcome data.

All included patients underwent level I–II axillary lymph node dissection. Clinical indications included clinically or radiologically suspicious axillary lymph nodes with metastasis confirmed by fine-needle aspiration or core-needle biopsy; clinically or pathologically confirmed residual axillary disease after neoadjuvant systemic therapy; or sentinel lymph node macrometastasis (>2 mm) in patients undergoing total mastectomy for whom multidisciplinary assessment indicated completion of axillary staging and regional nodal radiotherapy was not used as an alternative to axillary dissection.

All eligible cases during the study period were included, and no *a priori* sample-size calculation was performed. The study protocol was approved by the medical ethics committee of our hospital. Because only pre-existing clinical data were used, the requirement for written informed consent was waived. All data were de-identified before analysis, and the study was conducted in accordance with the principles of the Declaration of Helsinki.

### 2.2 Surgery, perioperative pathway, and drain management

All patients underwent modified radical mastectomy preserving the pectoralis major muscle with level I–II axillary lymph node dissection, performed by a dedicated breast surgical team according to a standardized technique. Monopolar electrocautery was the principal instrument used for flap dissection in all 306 patients; ultrasonic devices or other energy platforms were not used during the study period. During axillary dissection, visible lymphatic vessels and blood vessels were sealed with titanium clips, ligatures, or electrocautery. Before wound closure, the operative field was reinspected, and hemostasis was completed using electrocautery or ligation. No patient underwent flap fixation, quilting sutures, additional dead-space obliteration, application of fibrin sealant, or use of another tissue adhesive. After mastectomy and axillary dissection, one closed-suction drain was placed beneath the chest-wall flap and another in the axilla. Operations were performed by three attending surgeons, each with more than 10 years of experience in breast surgery. The core operative steps, drain configuration, and wound-closure principles remained unchanged from January 2021 through December 2024.

Perioperative antimicrobial prophylaxis consisted of cefazolin 2 g administered intravenously 30–60 min before skin incision; patients with β-lactam allergy received clindamycin 600 mg. Antimicrobial prophylaxis was discontinued within 24 h after surgery. After the incision had been covered with a sterile nonadherent dressing and absorbent pad, a broad elastic bandage was applied uniformly over the chest wall and axilla and maintained for 48 h. Skin-flap color, temperature, capillary refill, and patient discomfort were evaluated at 6 h postoperatively and daily thereafter; the bandage was loosened if flap perfusion appeared compromised or substantial pain occurred. Compression pressure was not quantified using a manometer, but all patients received the same materials, coverage area, duration, and monitoring procedure, and the protocol did not change during the study period. Pressure wound dressing with an elastic bandage may affect daily and total drainage volumes, and the degree of standardization may influence interpretation of drainage trajectories (13). Functional exercises for the ipsilateral upper limb began on postoperative day 1 and included active movements of the hand, wrist, and elbow. Shoulder flexion and abduction were restricted to ≤90° during postoperative days 1–3, after which the range of motion was gradually increased as tolerated; full-range activity was progressively resumed after drain removal. These protocols for antimicrobial prophylaxis, compression dressing, and ipsilateral upper-limb exercise were applied to all patients and remained unchanged throughout the study period.

Nurses summarized drainage volumes over consecutive 24-h intervals using time-stamped nursing records. Postoperative day 1 was defined as 0–24 h after completion of skin closure, with subsequent intervals recorded through 144–168 h. Daily total drainage was calculated as the sum of chest-wall and axillary drainage during the same interval and was denoted D1–D7, in mL/24 h. Drain removal was assessed after completion of the seventh 24-h recording interval. Both drains were removed when their combined output had remained <30 mL/24 h for 2 consecutive days and there was no active bleeding, purulent drainage, or drain dysfunction.

### 2.3 Clinical data, ascertainment of complications, and quality control

A standardized data-collection form was used to record age, BMI, smoking history, diabetes mellitus, American Society of Anesthesiologists (ASA) physical status classification (14), neoadjuvant systemic therapy, postoperative pathologic T category, molecular subtype, resected specimen weight, operative time, intraoperative blood loss, number of dissected lymph nodes, number of positive lymph nodes, time to drain removal, and postoperative complications. Pathologic T category (pT) was recorded for patients who had not received neoadjuvant therapy, whereas post-neoadjuvant pathologic T category (ypT) was recorded for those who had (15). Molecular subtype was determined according to hormone receptor (HR) and human epidermal growth factor receptor 2 (HER2) status (16).

Postoperative complication data were obtained from inpatient progress notes, nursing records, laboratory and microbiological results, imaging examinations, outpatient follow-up, readmissions, and records of repeat interventions and were followed through postoperative day 30. Surgical site infection was classified as superficial incisional or deep incisional infection occurring within 30 days after surgery. Superficial incisional infection involved the skin or subcutaneous tissue and met at least one of the following criteria: purulent drainage; a positive culture from an aseptically obtained specimen; deliberate opening of the incision accompanied by pain or tenderness, localized swelling, erythema, or heat; or diagnosis by a clinician. Deep incisional infection involved the fascia or muscle and met at least one of the following criteria: purulent drainage from the deep incision; spontaneous dehiscence or deliberate opening or aspiration accompanied by fever or localized pain or tenderness; or an abscess or infection confirmed by imaging, surgery, or histopathology (17). Hematoma was defined as a clinically or radiologically confirmed blood collection requiring aspiration, transfusion, surgical evacuation, or a change in wound-management strategy. Flap necrosis was defined as full-thickness necrosis of the skin or flap tissue requiring debridement, prolonged dressing changes, or reoperation. Early ipsilateral upper-extremity lymphedema/swelling was defined, within 30 days after surgery, as a circumference difference of ≥2 cm between the ipsilateral and contralateral arms at any prespecified site (the wrist crease, elbow crease, or 10 cm above the elbow) or an estimated ipsilateral limb-volume increase of ≥10% relative to the contralateral limb using the truncated-cone formula based on circumferences at adjacent levels. These thresholds were based on diagnostic research on lymphedema after breast cancer treatment (18), and an event was recorded only when both the measurement site and value were documented. “Other postoperative complications” was defined as the occurrence of at least one of these events within 30 days after surgery and was counted at the patient level.

Two uniformly trained investigators independently extracted the data and reviewed the inpatient, nursing, laboratory and microbiological, imaging, outpatient, readmission, and repeat-intervention records for all 306 patients on a case-by-case basis. Disagreements were reviewed and adjudicated by a third senior physician.

### 2.4 Identification of drainage-volume decline trajectories during the first 7 postoperative days

Daily drainage volumes were right-skewed. Before modeling, D1–D7 values were separately transformed using the natural logarithm, ln(drainage volume + 1). Latent class mixed-effects models were used to identify latent trajectories of early postoperative drainage volume over time, with postoperative day as the time variable and a patient-level random intercept included in the model.

Models with one to five latent classes were fitted sequentially, and linear, quadratic, and cubic time functions were evaluated for each class solution. For each number of classes, the time-function model with the lowest Bayesian information criterion (BIC) was selected as the candidate model. Models were repeatedly fitted using multiple sets of random initial values. The final model was selected by jointly considering the BIC, entropy ≥0.70, mean posterior class-membership probability ≥0.70 for each class, minimum class proportion ≥5%, and clinical interpretability. The 30-day outcome and clinical covariates were not included in trajectory identification, and each patient was assigned to the class with the highest posterior probability. Because D1–D7 and their derived measures contributed to class formation, these variables were presented descriptively without inferential comparisons among classes.

### 2.5 Outcome definition and adjudication

The primary outcome was persistent seroma on postoperative day 30. Patients underwent outpatient assessment on postoperative day 30 within an allowable window of ±3 days, and drain status on day 30 was determined from the actual drain-removal date. Persistent seroma was defined as a serous collection that persisted or recurred beneath the chest-wall flap or in the axilla on day 30 and met at least one of the following criteria: an ultrasonographically confirmed collection requiring needle aspiration; drain reinsertion because of persistent or recurrent fluid accumulation; or retention of the original drain on day 30 because of ongoing serous output. An ultrasonographically confirmed serous collection was considered to require aspiration when at least one of the following clinical conditions was documented: substantial pain or tension, pressure on the incision or flap, restricted upper-limb movement, progressive enlargement on serial assessment, or a need to obtain a fluid sample because of suspected infection. No fixed threshold for collection volume or maximum diameter was used; the decision to aspirate was based on the above clinical manifestations, effects on local tissue, and dynamic changes on serial assessment. Small, stable, asymptomatic collections were managed expectantly and were not counted as primary-outcome events. Recurrent seroma was defined as reaccumulation after the first aspiration requiring repeat aspiration or drain reinsertion. Drain reinsertion was used for rapid reaccumulation after aspiration or a persistent clinically significant collection. This adjudication framework was based on evidence concerning postoperative seroma management (19). Persistent drainage caused by infection, hematoma, or another nonserous process, as well as hematoma, abscess, chyle leak, and small asymptomatic collections not requiring clinical management, was excluded. The strict outcome required that the patient first meet the primary day-30 outcome and, within 30 postoperative days, undergo at least two aspirations in total or drain reinsertion because of persistent or recurrent fluid accumulation.

Outcome management was classified according to a mutually exclusive hierarchy, in the following order: drain reinsertion, original drain still in place on postoperative day 30, and needle aspiration only. Two physicians who were not involved in trajectory modeling independently adjudicated outcomes using outpatient records, ultrasonography results, aspiration or drain-reinsertion records, and drain-removal dates. They were blinded to trajectory class, and disagreements were adjudicated by a senior physician.

### 2.6 Statistical analysis

Statistical analyses were performed using R version 4.3.3. The distributions of continuous variables were evaluated using histograms, quantile–quantile plots, and tests of normality. Normally distributed data are presented as mean ± standard deviation, skewed data as median [interquartile range], and categorical variables as number and percentage. Across the three trajectory classes, normally distributed continuous variables were compared using one-way analysis of variance, skewed continuous variables using the Kruskal–Wallis test, and categorical variables using the Pearson *χ^2^* test. The Fisher–Freeman–Halton exact test was used when the expected count was <1 in any cell or <5 in >20% of cells. For demographic and tumor characteristics, the maximum absolute standardized mean difference (SMD) between any two classes was also reported, with |SMD|>0.10 indicating imbalance. All between-class tests were omnibus tests; no *post hoc* pairwise comparisons were performed.

Unadjusted odds ratios (ORs) were estimated using standard binary logistic regression. Adjusted associations were evaluated using Firth penalized-likelihood logistic regression, with the rapid-decline trajectory as the reference category. Covariates were prespecified before outcome modeling according to a clinical causal framework and included patient susceptibility factors (age, BMI, and diabetes mellitus), preoperative treatment (neoadjuvant systemic therapy), and operative extent or complexity (resected specimen weight, operative time, and number of dissected lymph nodes); they were not selected according to univariable *P* values. The ASA physical status classification integrates information on age, obesity, diabetes mellitus, and other comorbidities and therefore overlapped with the patient susceptibility factors already included. Given the parameter constraint imposed by 66 primary-outcome events, ASA physical status was not included as an additional parameter in the primary model. The pT/ypT category represented disease burden and was evaluated in a targeted extended sensitivity model. Time to drain removal and postoperative complications may lie on the causal pathway between exposure and outcome and were not included in the primary model. Results are reported as ORs and 95% confidence intervals (CIs). Variance inflation factors were used to assess multicollinearity, and restricted cubic splines were used to assess nonlinearity between continuous covariates and the log odds of the outcome.

The primary exposure and primary outcome were not imputed. Missing covariate data were handled using multiple imputation by chained equations, generating 20 datasets. The imputation model included all analysis variables, the same model was fitted in each dataset, and regression coefficients and variances were pooled according to Rubin’s rules.

Sensitivity analyses included posterior-probability-weighted regression with patient-level cluster-robust standard errors; complete-case analysis; use of the strict outcome definition, requiring the primary outcome plus at least two aspirations in total or drain reinsertion within 30 postoperative days because of persistent or recurrent fluid accumulation; use of an alternative outcome definition that excluded retention of the original drain on postoperative day 30, retained all 306 patients, and refitted Firth regression with the same seven prespecified covariates as in the primary model; separate addition of pT/ypT category and operating surgeon to the primary model, based on the 66 primary-outcome events; replacement of trajectory class by either 7-day cumulative drainage or the relative reduction from D1 to D7; and restricted cubic spline assessment of nonlinearity for continuous drainage measures.

To compare the information provided by trajectory classification with that provided by simpler drainage measures, standard multivariable logistic regression was used consistently among 289 complete cases to fit a clinical-covariate model; models adding D7 drainage, 7-day cumulative drainage, the relative reduction from D1 to D7, or trajectory class; and three extended models that added trajectory class to the D7 drainage, 7-day cumulative drainage, and D1-to-D7 relative-reduction models, respectively. Relative performance was compared using the Akaike information criterion (AIC), Brier score, and C statistic. The AIC, Brier score, and C statistic were treated as apparent performance estimates among the 289 complete cases. Likelihood-ratio tests were used for the three nested model comparisons. Differences in the C statistic after adding trajectory class and their 95% CIs were estimated using 1, 000 patient-level nonparametric bootstrap resamples, with each model refitted in every resample. No optimism correction, external validation, or assessment of clinical net benefit was performed. All tests were two-sided, and *P* < 0.05 was considered statistically significant.

## 3 Results

### 3.1 Study population and data completeness

A total of 345 patients were screened. Twelve patients who underwent bilateral surgery or simultaneous breast reconstruction, six with previous ipsilateral breast or axillary surgery or radiotherapy, four with distant metastasis before surgery, seven who underwent reoperation within 7 postoperative days or had drain malfunction, six with incomplete D1–D7 records, and four with missing 30-day outcome data were excluded, leaving 306 patients in the final analysis. D1–D7 drainage data and 30-day outcomes were complete for all patients. Among covariates, BMI was missing for six patients, resected specimen weight for five, operative time for three, and number of dissected lymph nodes for three; 17 patients had at least one missing covariate. The complete-case analysis included 289 patients, of whom 61 experienced the primary outcome. The primary adjusted analysis used 20 multiply imputed datasets.

Postoperative complication data for all 306 patients underwent case-by-case medical-record review. Discrepancies regarding the type or date of a complication were identified in six patients and were adjudicated by a third senior physician. Ultimately, 29 patients experienced at least one prespecified complication: six in the rapid-decline class, 13 in the moderate-decline class, and 10 in the persistently high-output class. During independent adjudication of the primary day-30 outcome, all six initial disagreements were resolved by the third senior physician.

### 3.2 Drainage-volume decline trajectories during the first 7 postoperative days

#### 3.2.1 Trajectory-model selection

The three-class model with a linear time function had the lowest BIC. Entropy and mean posterior class-membership probabilities for all classes exceeded the prespecified thresholds, and the minimum class proportion also met the prespecified criterion. The smallest classes in the four- and five-class models accounted for <5% of the sample; therefore, the three-class model was selected (**Table 1**).

**Table 1:** Fit indices for latent class models of drainage volume during postoperative days 1-7.

Number of classes	BIC	Entropy	Class composition, *n* (%)	Smallest class, %	Range of mean posterior probabilities	Prespecified criteria met
1	2101.45	—	306 (100.0)	100.0	—	No latent classes identified
2	331.51	0.9996	123 (40.2)/183 (59.8)	40.2	0.9999-1.0000	BIC higher than that of the three-class model
3	−74.89	0.9829	123 (40.2)/127 (41.5)/ 56 (18.3)	18.3	0.9782-0.9999	Yes
4	−42.43	0.9584	123 (40.2)/114 (37.3)/ 13 (4.2)/56 (18.3)	4.2	0.7995-1.0000	Smallest class <5%
5	−27.34	0.9800	120 (39.2)/3 (1.0)/5 (1.6)/ 122 (39.9)/56 (18.3)	1.0	0.9035-0.9980	Smallest class <5%

BIC, Bayesian information criterion; lower values indicate better relative fit among candidate models. *n* denotes the number of patients and % denotes percentage. Entropy measures class separation, with values closer to 1 indicating greater classification certainty. Mean posterior probability is the average posterior probability of membership in the assigned class among patients assigned to that class. “—” indicates not applicable. For each number of classes, the table presents the time-function model with the lowest BIC. Class composition is ordered from the lowest to the highest model-predicted drainage volume on postoperative day 7.

#### 3.2.2 Drainage characteristics of the trajectory classes

According to maximum posterior probability, 123 patients were assigned to the rapid-decline class, 127 to the moderate-decline class, and 56 to the persistently high-output class. Daily total drainage decreased in all three groups from D1 to D7. The rapid-decline class had the lowest daily levels and the greatest decline, the moderate-decline class had intermediate values, and the persistently high-output class had the highest daily levels and the smallest decline. Seven-day cumulative drainage increased progressively from the rapid-decline to the persistently high-output class, whereas the relative reduction from D1 to D7 decreased progressively (**Table 2**; **Figure 1**).

**Table 2:** Drainage characteristics during postoperative days 1–7 according to drainage-volume decline trajectory.

Measure	All patients (*n* = 306)	Rapid-decline trajectory (*n* = 123)	Moderate-decline trajectory (*n* = 127)	Persistently high-output trajectory (*n* = 56)
D1, mL/24 h	273 [222, 354]	243 [202, 300]	287 [222, 345]	353 [260, 414]
D2, mL/24 h	212 [158, 270]	161 [124, 200]	221 [187, 280]	290 [257, 340]
D3, mL/24 h	156 [112, 216]	106 [83, 131]	180 [146, 225]	265 [209, 333]
D4, mL/24 h	116 [75, 179]	68 [56, 81]	140 [114, 179]	240 [190, 303]
D5, mL/24 h	95 [52, 142]	43 [37, 57]	116 [97, 134]	230 [184, 281]
D6, mL/24 h	74 [33, 121]	29 [24, 37]	91 [74, 110]	208 [158, 268]
D7, mL/24 h	59 [21, 100]	20 [16, 23]	76 [60, 93]	189 [153, 246]
7-day cumulative drainage, mL	1000 [711, 1364]	668 [567, 834]	1119 [918, 1340]	1820 [1438, 2171]
Relative reduction from D1 to D7, %	77.5 [65.7, 91.9]	92.6 [91.1, 93.6]	73.2 [68.0, 77.4]	44.4 [38.0, 50.9]

Data are presented as median [interquartile range (IQR)]. *n* denotes the number of patients, mL denotes milliliters, and h denotes hours. D1–D7 denote the combined chest-wall and axillary drainage volumes during the first through seventh consecutive 24-h postoperative intervals. Because these measures were used to form the trajectory classes, they are presented descriptively and were not subjected to inferential comparisons among classes.

**Figure 1:**
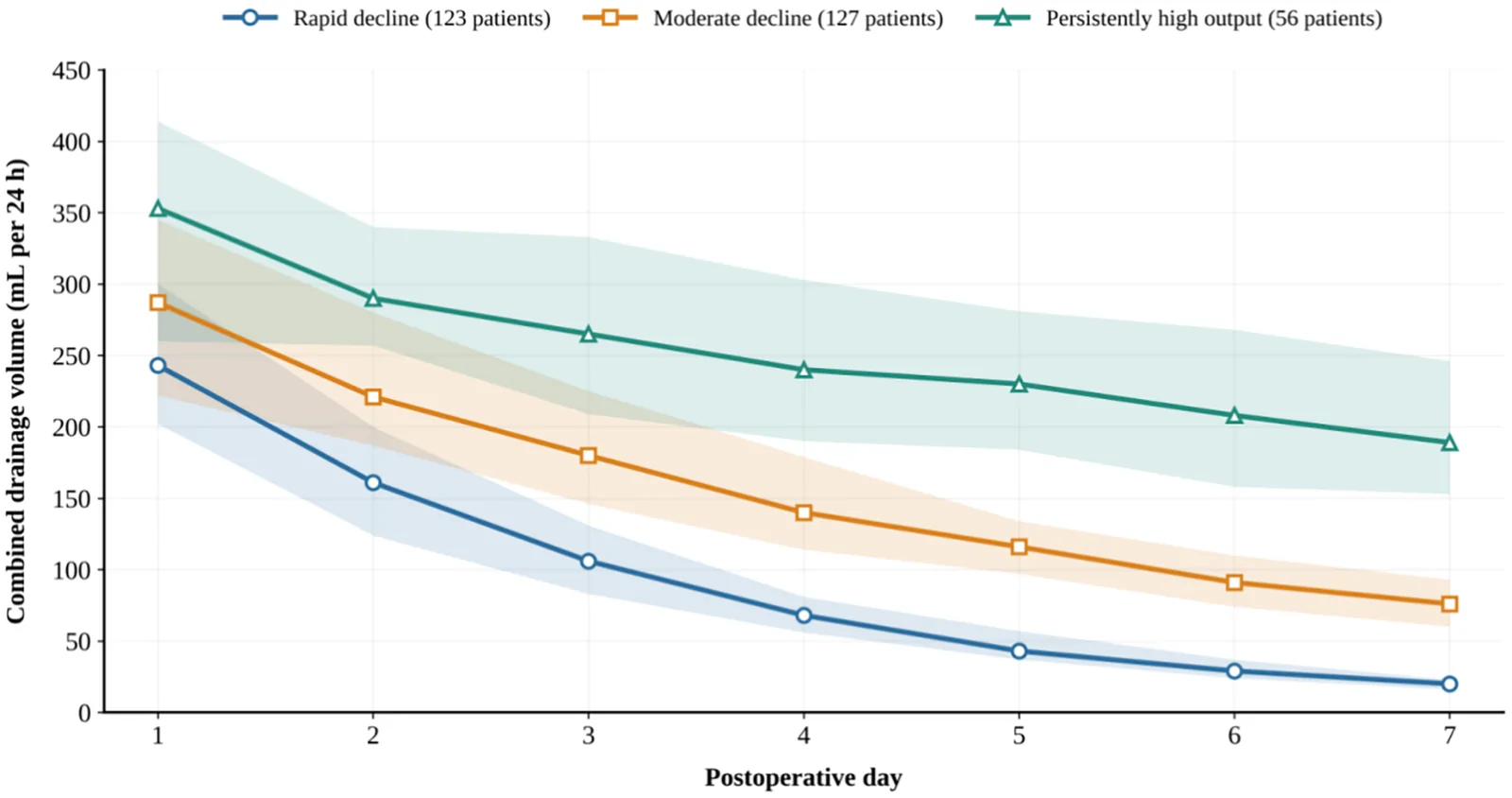
Drainage trajectories during the first 7 days after modified radical mastectomy with level I–II axillary lymph node dissection for breast cancer. Solid lines represent the median daily combined chest-wall and axillary drainage volume in each trajectory class, and shaded bands represent the interquartile range. The rapid-decline, moderate-decline, and persistently high-output classes included 123, 127, and 56 patients, respectively. D1–D7 denote the first through seventh consecutive 24-h postoperative intervals; mL denotes milliliters and h denotes hours.

### 3.3 Demographic and tumor pathologic characteristics

Age and the distribution of molecular subtypes were similar among the three groups (all *P*>0.05). From the rapid-decline to the persistently high-output class, BMI and the proportions of patients with a smoking history, diabetes mellitus, ASA class III status, and neoadjuvant systemic therapy generally increased; however, the omnibus differences were not statistically significant (all *P*>0.05). The persistently high-output class had a lower proportion of pT1/ypT1 disease and higher proportions of pT2/ypT2 and pT3–4/ypT3–4 disease. The overall distribution of postoperative pathologic T category differed significantly among the classes (*P* = 0.005). Except for molecular subtype, several characteristics had a maximum |SMD|>0.10, indicating varying degrees of imbalance (**Table 3**).

**Table 3:** Demographic and tumor pathologic characteristics according to drainage-volume decline trajectory.

Characteristic	All patients (*n* = 306)	Rapid-decline (*n* = 123)	Moderate-decline (*n* = 127)	Persistently high-output (*n* = 56)	Statistic	*P* value	Maximum |SMD|
Age, years	54.7 ± 11.0	54.0 ± 10.8	55.0 ± 10.7	55.3 ± 12.1	*F*(2, 303) = 0.38	0.687	0.11
Body mass index, kg/m^2^	24.2 ± 3.1	23.8 ± 3.1	24.3 ± 3.1	25.0 ± 3.1	*F*(2, 297) = 2.85	0.060	0.39
Smoking history, *n* (%)	30 (9.8)	10 (8.1)	13 (10.2)	7 (12.5)	*χ^2^*(2) = 0.88	0.645	0.14
Diabetes mellitus, *n* (%)	33 (10.8)	9 (7.3)	14 (11.0)	10 (17.9)	*χ^2^*(2) = 4.46	0.108	0.32
ASA physical status, *n* (%)					*χ^2^*(4) = 8.83	0.066	0.37
I	108 (35.3)	52 (42.3)	42 (33.1)	14 (25.0)	—	—	—
II	175 (57.2)	65 (52.8)	76 (59.8)	34 (60.7)	—	—	—
III	23 (7.5)	6 (4.9)	9 (7.1)	8 (14.3)	—	—	—
Neoadjuvant systemic therapy, *n* (%)	70 (22.9)	21 (17.1)	31 (24.4)	18 (32.1)	*χ^2^*(2) = 5.24	0.073	0.36
Postoperative pathologic T category, *n* (%)					*χ^2^*(4) = 14.74	0.005	0.59
pT1/ypT1	125 (40.8)	62 (50.4)	50 (39.4)	13 (23.2)	—	—	—
pT2/ypT2	152 (49.7)	52 (42.3)	67 (52.8)	33 (58.9)	—	—	—
pT3–4/ypT3–4	29 (9.5)	9 (7.3)	10 (7.9)	10 (17.9)	—	—	—
Molecular subtype, *n* (%)					*χ^2^*(6) = 0.87	0.990	0.09
HR-positive/HER2-negative	204 (66.7)	82 (66.7)	85 (66.9)	37 (66.1)	—	—	—
HR-positive/HER2-positive	32 (10.5)	12 (9.8)	15 (11.8)	5 (8.9)	—	—	—
HR-negative/HER2-positive	27 (8.8)	12 (9.8)	10 (7.9)	5 (8.9)	—	—	—
Triple-negative	43 (14.1)	17 (13.8)	17 (13.4)	9 (16.1)	—	—	—

Normally distributed continuous variables are presented as mean ± standard deviation, and categorical variables as *n* (%). *n* denotes the number of patients; % denotes percentage; and kg/m^2^ denotes kilograms per square meter. *F* is the one-way analysis of variance statistic, *χ^2^* is the Pearson chi-square statistic, *P* is the two-sided *P* value, and “—” indicates not applicable. Body mass index data were available for 300 patients: 123, 124, and 53 in the rapid-decline, moderate-decline, and persistently high-output classes, respectively. ASA denotes American Society of Anesthesiologists physical status; pT, pathologic T category; ypT, post-neoadjuvant pathologic T category; HR, hormone receptor; HER2, human epidermal growth factor receptor 2; and SMD, standardized mean difference. Maximum |SMD| is the largest absolute standardized difference between any two trajectory classes.

### 3.4 Operative and postoperative characteristics

Resected specimen weight, operative time, intraoperative blood loss, and number of dissected lymph nodes increased progressively from the rapid-decline to the persistently high-output class (all *P* < 0.001), as did the number of positive lymph nodes (*P* = 0.015). Time to drain removal increased in the same order (*P* < 0.001), and the incidence of other postoperative complications also increased (*P* = 0.021) (**Table 4**).

**Table 4:** Operative and postoperative characteristics according to drainage-volume decline trajectory.

Characteristic	All patients (*n* = 306)	Rapid-decline (*n* = 123)	Moderate-decline (*n* = 127)	Persistently high-output (*n* = 56)	Statistic	*P* value
Resected specimen weight, g	571 [462, 723]	504 [428, 592]	590 [497, 707]	735 [604, 941]	*H*(2) = 55.64	<0.001
Operative time, min	115.3 ± 19.2	108.6 ± 16.7	116.0 ± 18.9	128.6 ± 17.9	*F*(2, 300) = 23.93	<0.001
Intraoperative blood loss, mL	77 [58, 104]	65 [52, 82]	82 [60, 106]	102 [81, 122]	*H*(2) = 45.12	<0.001
Number of dissected lymph nodes	15.6 ± 4.3	14.2 ± 4.0	15.7 ± 4.0	18.6 ± 4.5	*F*(2, 300) = 22.11	<0.001
Number of positive lymph nodes	1 [0, 2]	1 [0, 2]	1 [0, 2]	2 [0, 3]	*H*(2) = 8.39	0.015
Time to drain removal, postoperative days	10.0 [8.0, 12.0]	8.0 [7.5, 9.0]	11.0 [10.0, 12.0]	16.5 [15.0, 19.0]	*H*(2) = 228.90	<0.001
Other postoperative complications, *n* (%)	29 (9.5)	6 (4.9)	13 (10.2)	10 (17.9)	*χ^2^*(2) = 7.70	0.021

Normally distributed continuous variables are presented as mean ± standard deviation, skewed continuous variables as median [interquartile range (IQR)], and categorical variables as *n* (%). *n* denotes the number of patients; % denotes percentage; g, grams; min, minutes; and mL, milliliters. *F* is the one-way analysis of variance statistic, *H* is the Kruskal–Wallis statistic, *χ^2^* is the Pearson chi-square statistic, and *P* is the two-sided *P* value. Resected specimen weight was available for 301 patients: 120, 125, and 56 in the rapid-decline, moderate-decline, and persistently high-output classes, respectively. Operative time was available for 303 patients: 122, 126, and 55, respectively. The number of dissected lymph nodes was available for 303 patients: 121, 126, and 56, respectively. Time to drain removal was influenced by the prespecified removal criterion and is reported as a descriptive clinical variable.

### 3.5 Persistent seroma on postoperative day 30

Among 306 patients, 66 developed persistent seroma on postoperative day 30, for an overall incidence of 21.6%. Events occurred in 9, 25, and 32 patients in the rapid-decline, moderate-decline, and persistently high-output classes, respectively, and the incidence increased across classes (omnibus *P* < 0.001). Among the 66 patients with an outcome event, 48 underwent needle aspiration only, nine underwent drain reinsertion, and nine still had the original drain in place on day 30. The distribution of these three management modalities did not differ significantly among trajectory classes (*P* = 0.974) (**Table 5**).

**Table 5:** Persistent seroma on postoperative day 30 and management modality according to drainage-volume decline trajectory.

Outcome	All patients	Rapid-decline	Moderate-decline	Persistently high-output	Statistic	*P* value
Persistent seroma, events/total (%)	66/306 (21.6)	9/123 (7.3)	25/127 (19.7)	32/56 (57.1)	*χ^2^*(2) = 56.93	<0.001
Management modality, *n*/outcome events (%)					*χ^2^*(4) = 0.51†	0.974
Needle aspiration only	48/66 (72.7)	7/9 (77.8)	19/25 (76.0)	22/32 (68.8)	—	—
Drain reinsertion	9/66 (13.6)	1/9 (11.1)	3/25 (12.0)	5/32 (15.6)	—	—
Original drain still in place on postoperative day 30	9/66 (13.6)	1/9 (11.1)	3/25 (12.0)	5/32 (15.6)	—	—

*n* denotes the number of patients, % denotes percentage, *χ^2^* is the Pearson chi-square statistic, *P* is the two-sided *P* value, and “—” indicates not applicable. Management modalities were assigned using the following mutually exclusive hierarchy: drain reinsertion, original drain still in place on postoperative day 30, and needle aspiration only. †Because several cells had expected counts <5, the *P* value for the omnibus comparison of management modality was obtained using the Fisher–Freeman–Halton exact test; the Pearson *χ^2^* statistic is also shown.

### 3.6 Association between drainage-volume decline trajectory and persistent seroma

With the rapid-decline trajectory as the reference category, the unadjusted ORs for the moderate-decline and persistently high-output trajectories were 3.10 (95% CI, 1.38-6.96; *P* = 0.006) and 16.89 (95% CI, 7.14-39.94; *P* < 0.001), respectively. After adjustment for the prespecified covariates, the corresponding ORs were 2.58 (95% CI, 1.10-6.07; *P* = 0.030) and 11.44 (95% CI, 3.92-33.37; *P* < 0.001), respectively (**Table 6**; **Figure 2**). Variance inflation factors ranged from 1.02 to 1.92, and tests for nonlinearity of continuous covariates were not statistically significant (all *P*>0.05).

**Table 6:** Logistic regression analysis of the association between drainage-volume decline trajectory and persistent seroma on postoperative day 30.

Trajectory class	Unadjusted OR (95% CI)	Wald *z*	*P* value	Adjusted OR (95% CI)	Wald *z*	*P* value
Rapid-decline	1.00 (reference)	—	—	1.00 (reference)	—	—
Moderate-decline	3.10 (1.38-6.96)	2.75	0.006	2.58 (1.10-6.07)	2.18	0.030
Persistently high-output	16.89 (7.14-39.94)	6.44	<0.001	11.44 (3.92-33.37)	4.46	<0.001

OR denotes odds ratio; CI, confidence interval; *z*, Wald *z* statistic; *P*, two-sided *P* value; and “—”, reference category or not applicable. The unadjusted model used standard binary logistic regression. The adjusted model used Firth penalized-likelihood logistic regression and included age, body mass index, diabetes mellitus, neoadjuvant systemic therapy, resected specimen weight, operative time, and number of dissected lymph nodes. Adjusted estimates were pooled across 20 multiply imputed datasets according to Rubin’s rules; Wald *z* statistics were based on large-sample approximations.

**Figure 2:**
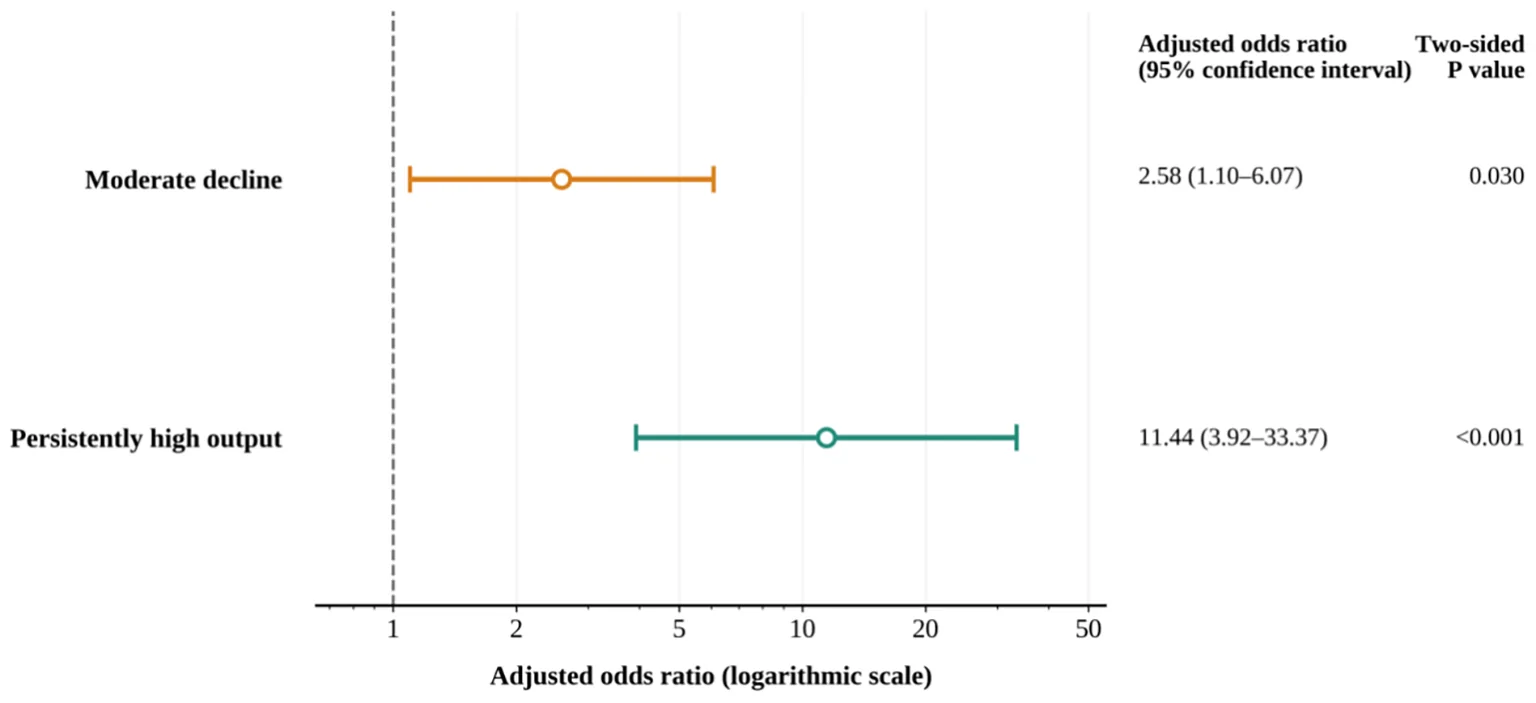
Adjusted association between drainage-volume decline trajectory and persistent seroma on postoperative day 30. With the rapid-decline trajectory as the reference category, points represent adjusted odds ratios and horizontal lines represent 95% confidence intervals. The horizontal axis is logarithmic. The model was adjusted for age, body mass index, diabetes mellitus, neoadjuvant systemic therapy, resected specimen weight, operative time, and number of dissected lymph nodes.

### 3.7 Sensitivity analyses

After posterior-probability weighting, the adjusted ORs for the moderate-decline and persistently high-output trajectories were 2.57 (95% CI, 1.08–6.09; *P* = 0.032) and 11.32 (95% CI, 3.91–32.75; *P* < 0.001), respectively. In the complete-case analysis, the corresponding ORs were 2.43 (95% CI, 0.98–6.01; *P* = 0.055) and 12.60 (95% CI, 4.07–39.03; *P* < 0.001). Under the strict outcome definition—requiring the primary day-30 outcome plus at least two aspirations in total or drain reinsertion within 30 postoperative days because of persistent or recurrent fluid accumulation—46 patients met the outcome criterion: five in the rapid-decline class, 15 in the moderate-decline class, and 26 in the persistently high-output class. The corresponding ORs were 2.73 (95% CI, 0.92–8.11; *P* = 0.070) and 14.70 (95% CI, 4.14–52.18; *P* < 0.001).

After exclusion of the original-drain-retention component on postoperative day 30, 57 patients met the alternative outcome definition: eight in the rapid-decline class, 22 in the moderate-decline class, and 27 in the persistently high-output class. After adjustment for the same seven prespecified covariates as in the primary model, the ORs were 2.45 (95% CI, 1.01-5.94; *P* = 0.047) for the moderate-decline class and 9.21 (95% CI, 3.11-27.28; *P* < 0.001) for the persistently high-output class. In extended models based on the 66 primary-outcome events, additional adjustment for pT/ypT category yielded corresponding ORs of 2.42 (95% CI, 1.02-5.76; *P* = 0.045) and 9.78 (95% CI, 3.31-28.90; *P* < 0.001), whereas additional adjustment for operating surgeon yielded ORs of 2.56 (95% CI, 1.08-6.06; *P* = 0.033) and 11.18 (95% CI, 3.81-32.79; *P* < 0.001).

When continuous measures replaced trajectory class, the adjusted OR was 1.12 (95% CI, 1.06-1.19; *P* < 0.001) for each 100-mL increase in 7-day cumulative drainage and 1.51 (95% CI, 1.25-1.82; *P* < 0.001) for each 10-percentage-point decrease in the relative reduction from D1 to D7. Tests for nonlinearity were not statistically significant (all *P*>0.05). The persistently high-output trajectory retained a strong and directionally consistent association across sensitivity analyses. By contrast, the 95% CIs for the moderate-decline trajectory crossed 1 in the complete-case and strict-outcome analyses, indicating less stable evidence of association (**Supplementary Table 1**).

### 3.8 Incremental information of trajectory classification beyond simpler drainage measures

Among 289 complete cases, all models that incorporated drainage information had lower AIC and Brier scores and higher C statistics than the clinical-covariate model in the current sample. The trajectory-class model and D7-drainage model showed the most similar relative performance. The 7-day cumulative-drainage model showed intermediate performance, whereas the D1-to-D7 relative-reduction model improved on the clinical model but performed less well than the trajectory-class and D7 models. After trajectory class was added to the corresponding simpler-measure models, the likelihood-ratio test for the D7-drainage model was *χ^2^*(2)=5.00 (*P* = 0.082), and the C statistic increased by 0.008 (95% CI, −0.004 to 0.020; *P* = 0.191). For the 7-day cumulative-drainage model, the likelihood-ratio test was *χ^2^*(2)=10.60 (*P* = 0.005), and the C statistic increased by 0.021 (95% CI, 0.006–0.036; *P* = 0.006). For the D1-to-D7 relative-reduction model, the likelihood-ratio test was *χ^2^*(2)=19.30 (*P* < 0.001), and the C statistic increased by 0.043 (95% CI, 0.025–0.061; *P* < 0.001). Trajectory class provided additional information beyond 7-day cumulative drainage and relative reduction but offered only a small increment beyond D7 drainage, with limited statistical evidence. All performance measures were apparent estimates in the current sample (**Supplementary Table 2**).

## 4 Discussion

Using serial drainage records from the first 7 postoperative days, this study identified rapid-decline, moderate-decline, and persistently high-output trajectories. Although the persistently high-output class comprised only 18.3% of the cohort, its incidence of persistent seroma on postoperative day 30 was 57.1%. The association remained strong after accounting for class-membership uncertainty, restricting the analysis to complete cases, applying a strict outcome definition, excluding the prolonged original-drain component, and additionally adjusting for pT/ypT category and operating surgeon. The moderate-decline trajectory was associated with the outcome in the primary model, but its 95% CI crossed 1 in the complete-case and strict-outcome analyses, indicating less stable evidence than that for the persistently high-output trajectory. These findings suggest that a persistently high-output pattern may represent a dynamic postoperative risk phenotype warranting further validation.

The standardized use of two closed-suction drains reduced between-group variation attributable to drainage-device configuration. Recent clinical data suggest that single- versus double-drain configurations may affect patient comfort, total drainage volume, and time to drain removal, although their effects on seroma are not fully consistent (20). Another prospective study likewise showed that changing the number of drains did not fully explain the risk of persistent fluid accumulation (21). The trajectory differences observed in this study may jointly reflect fluid production, dead-space closure, and changes in lymphatic return over time; nevertheless, effects of unmeasured operative factors cannot be excluded.

Dead-space management is an important modifiable determinant of seroma. Recent evidence indicates that suture fixation is superior to tissue glue for reducing clinically significant seroma (22). Evidence specific to modified radical mastectomy with level I–II axillary dissection indicates that quilting sutures reduce seroma, aspiration requirements, and total drainage volume (23). Broader evidence likewise suggests relatively consistent effects of quilting sutures and flap fixation (24). The extent of quilting and technical details may influence effectiveness (25), whereas clinical acceptance is also affected by additional operative time, learning curves, and concerns regarding local tissue tension (26). No patient in the present study underwent flap fixation, quilting sutures, additional dead-space obliteration, or application of tissue adhesives; therefore, the trajectories reflect drainage changes under a uniform conventional surgical pathway. Residual confounding from electrocautery intensity, details of lymphatic control, and flap thickness remains possible.

The evidence supporting tissue adhesives is less consistent than that supporting quilting sutures or flap fixation. Recent evidence on TissuGlu did not demonstrate a consistent reduction in seroma and suggested a possible increase in overall seroma incidence (27). Direct comparative data support suture flap fixation for reducing the burden of fluid accumulation (28), whereas comparisons between fibrin glue and quilting indicate that effects vary according to material, application technique, and outcome definition (29). Tissue adhesives are therefore more appropriately considered investigational adjuncts, and routine use requires more consistent clinical evidence.

Pharmacologic and local biological pathways may further influence drainage trajectories. Recent data suggest that tranexamic acid can reduce drainage volume in some patients, although results for seroma endpoints remain inconsistent (30). Broader evidence similarly indicates a clearer effect on hematoma than on seroma (31). Perioperative corticosteroids may reduce local exudation, but dosing regimens and safety parameters require standardization (32). Changes in T-helper 2/T-helper 17 (Th2/Th17)-associated cytokines in seroma fluid support a relationship between persistent fluid accumulation and local immune responses (33), providing a potential biological explanation for the persistently high-output trajectory.

The day-30 composite endpoint included persistent seroma requiring clinical management and prolonged retention of the original drain. A radiologically detected collection that was small, stable, and asymptomatic was managed expectantly. Symptomatic seroma was characterized by pain, tension, pressure on the flap, or restricted movement. Seroma requiring aspiration had both ultrasonographic evidence and a clinical indication for intervention. Reaccumulation after the first aspiration that required repeat management was classified as recurrent seroma. Rapid reaccumulation after aspiration or a persistent clinically significant collection could require drain reinsertion, whereas an original drain remaining in place on day 30 reflected prolonged drainage. Among the 66 patients with an outcome event, 48 underwent needle aspiration only, nine underwent drain reinsertion, and nine had prolonged retention of the original drain, demonstrating that the components differed in clinical severity. Previous systematic reviews have also shown that heterogeneity in seroma definitions and intervention thresholds can substantially affect reported incidence (24). The strict outcome required both the primary day-30 outcome and at least two aspirations in total or drain reinsertion within 30 postoperative days. The persistently high-output trajectory remained strongly associated under this definition, although fewer events widened the CIs. Long-term data indicate that approximately 1.7% of patients after mastectomy undergo capsulectomy for chronic encapsulated seroma, and surgical management may still be followed by persistent fluid accumulation, infection, reoperation, and limited symptom relief (34). The 21.6% incidence observed in this study represents a specific intervention-related composite endpoint on postoperative day 30. Persistent fluid on day 30 is not equivalent to long-term refractory seroma, and this study did not observe subsequent resolution or long-term treatment courses.

Potentially high-risk patients may be identified using preoperative candidate factors, intraoperative/perioperative information, and postoperative dynamic phenotypes. Candidate preoperative factors include higher BMI, diabetes mellitus, neoadjuvant systemic therapy, imaging-estimated breast volume or tumor burden, planned total mastectomy, and the anticipated extent of axillary dissection. Intraoperative/perioperative information includes actual resected specimen weight, operative time, and the number of dissected lymph nodes. During the first 7 postoperative days, trajectory classification, D7 drainage, 7-day cumulative drainage, and the relative reduction from D1 to D7 may be evaluated as alternative measures for research-oriented risk stratification. The clinical benefit of adding trajectory classification to D7 drainage has not been established. Recent evidence supports further evaluation of standardized quilting sutures, flap fixation, dead-space management, and consistency of perioperative pathways (35). Pooled evidence also supports prioritizing flap fixation for further evaluation as a preventive strategy (36). In research-defined high-risk groups, intensified follow-up after discharge, earlier review, and selective ultrasonographic surveillance may be evaluated prospectively. The utility of alternative drain-removal thresholds, duration of drain retention, and preventive interventions likewise requires prospective comparison.

Trajectory analysis preserves longitudinal information from D1 to D7 and can describe dynamic phenotypes such as persistently high output, slow decline, and rapid decline. Exploratory model comparisons showed similar discrimination for the trajectory and D7 models. Adding trajectory class to the D7 model produced only a small increase in discrimination, with a 95% CI that included zero; the improvement in model fit was not statistically significant. In contrast, adding trajectory class to the 7-day cumulative-drainage and D1-to-D7 relative-reduction models improved model fit and discrimination. These results suggest that D7 drainage already contains substantial outcome-related information and that the incremental value of trajectory classification varies according to the simpler comparator. Trajectory classification should remain a research-oriented risk-stratification variable. The incremental clinical value of trajectory classification over the simpler D7 drainage measurement remains uncertain and requires prospective validation. Whether it can optimize observation, review, or ultrasonographic surveillance requires external validation, assessment of class-assignment reproducibility, assessment of discrimination and calibration, identification of clinically meaningful thresholds, evaluation of net benefit, and prospective testing of clinical utility.

This study has several limitations. First, the data were obtained from a single institution, and all patients underwent modified radical mastectomy with level I–II axillary lymph node dissection. External applicability is therefore mainly limited to similar surgical populations; patients undergoing sentinel lymph node biopsy, breast-conserving surgery, or immediate reconstruction require separate validation. Second, retrospective medical and nursing records may contain measurement errors in drainage volume and differences in clinical judgment. Although complication data were reviewed case by case, undocumented events may still have caused misclassification. Third, the primary outcome included retention of the original drain on postoperative day 30 and was therefore influenced by the prespecified drain-removal threshold, creating a risk of incorporation bias. Results remained directionally consistent after exclusion of this component and adjustment for the same covariates, with the persistently high-output trajectory showing the most stable association; however, effects of clinical management decisions remain possible. Fourth, the primary model limited the number of parameters according to a causal framework and the number of outcome events. ASA physical status, unquantified disease burden, electrocautery intensity, details of lymphatic control, flap thickness, and the actual pressure of compression dressings may have caused residual confounding. Fifth, the day-30 endpoint reflected early persistence and could not estimate repeated aspiration, drain reinsertion, infection, chronic capsule formation, reoperation, or delayed adjuvant treatment at 90 days or over longer periods. Sixth, the exploratory model comparison was based on 289 complete cases, and model performance represented apparent estimates in the current sample without optimism correction, external calibration, or assessment of clinical net benefit.

Future studies should use multicenter prospective designs, standardize surgical technique, compression dressing, drain removal, and aspiration criteria, and follow patients for 90 days to 6 months. In addition to fluid status on day 30, studies should record the number of aspirations, cumulative aspirated volume, drain reinsertion, infection, chronic capsule formation, reoperation, unplanned visits, delayed adjuvant treatment, and patient-reported outcomes. They should also test the reproducibility of trajectory classes across centers and operative approaches, compare the external discrimination and calibration of trajectories with simpler measures such as D7 drainage, and evaluate the clinical net benefit of trajectory-guided follow-up or intervention.

## 5 Conclusions

During the first 7 days after modified radical mastectomy with level I–II axillary lymph node dissection for breast cancer, drainage volume followed three decline trajectories. The persistently high-output trajectory showed the strongest and most consistent association with persistent seroma on postoperative day 30, whereas the moderate-decline trajectory was associated with the outcome in the primary model but showed less stable evidence across sensitivity analyses. Trajectory classification should remain a research-oriented postoperative risk-stratification variable. Exploratory comparisons showed limited incremental information beyond D7 drainage, although trajectory classification provided additional information beyond 7-day cumulative drainage and the D1-to-D7 relative reduction. Clinical application requires multicenter external validation using standardized long-term outcomes, assessment of class-assignment reproducibility, calibration, clinically meaningful thresholds, and prospective evaluation of utility. The incremental clinical value of trajectory classification over the simpler D7 drainage measurement remains uncertain and requires prospective validation.

## Data Availability

The raw data supporting the conclusions of this article will be made available by the authors, without undue reservation.

## Supplementary material

Supplementary Table 1 Sensitivity analyses of the association between drainage-volume decline trajectory and persistent seroma on postoperative day 30.

Supplementary Table 2 Exploratory comparison of trajectory classification with simpler drainage measures.
