# Supplementary material for: Association of drainage volume decline trajectories during the first 7 postoperative days with persistent seroma on postoperative day 30 after modified radical mastectomy for breast cancer
Source: Front Oncol. 2026 Oct 28;16:1981532. doi: 10.3389/fonc.2026.1981532 (PMC13751509; doi:10.3389/fonc.2026.1981532)
Supplement: Supplementary file 1 [file Supplementaryfile1.docx]

**Supplementary Material**

**Supplementary Table 1. Sensitivity analyses of the association between drainage-volume decline trajectory and persistent seroma on postoperative day 30**

| **Analysis** | **Exposure comparison or effect scale** | **Events/sample size** | **Effect estimate (95% CI)** | **Test statistic** | ***P* value** |
| --- | --- | --- | --- | --- | --- |
| Posterior-probability weighting | Moderate-decline vs rapid-decline | 66/306 | Adjusted OR 2.57 (1.08-6.09) | *z*=2.14 | 0.032 |
| Posterior-probability weighting | Persistently high-output vs rapid-decline | 66/306 | Adjusted OR 11.32 (3.91-32.75) | *z*=4.48 | <0.001 |
| Complete-case analysis | Moderate-decline vs rapid-decline | 61/289 | Adjusted OR 2.43 (0.98-6.01) | *z*=1.92 | 0.055 |
| Complete-case analysis | Persistently high-output vs rapid-decline | 61/289 | Adjusted OR 12.60 (4.07-39.03) | *z*=4.39 | <0.001 |
| Strict outcome definition | Moderate-decline vs rapid-decline | 46/306 | Adjusted OR 2.73 (0.92-8.11) | *z*=1.81 | 0.070 |
| Strict outcome definition | Persistently high-output vs rapid-decline | 46/306 | Adjusted OR 14.70 (4.14-52.18) | *z*=4.16 | <0.001 |
| Exclusion of original-drain-retention component | Moderate-decline vs rapid-decline | 57/306 | Adjusted OR 2.45 (1.01-5.94) | *z*=1.98 | 0.047 |
| Exclusion of original-drain-retention component | Persistently high-output vs rapid-decline | 57/306 | Adjusted OR 9.21 (3.11-27.28) | *z*=4.01 | <0.001 |
| Additional adjustment for pT/ypT | Moderate-decline vs rapid-decline | 66/306 | Adjusted OR 2.42 (1.02-5.76) | *z*=2.00 | 0.045 |
| Additional adjustment for pT/ypT | Persistently high-output vs rapid-decline | 66/306 | Adjusted OR 9.78 (3.31-28.90) | *z*=4.13 | <0.001 |
| Additional adjustment for operating surgeon | Moderate-decline vs rapid-decline | 66/306 | Adjusted OR 2.56 (1.08-6.06) | *z*=2.14 | 0.033 |
| Additional adjustment for operating surgeon | Persistently high-output vs rapid-decline | 66/306 | Adjusted OR 11.18 (3.81-32.79) | *z*=4.40 | <0.001 |
| Continuous-exposure analysis | Per 100-mL increase in 7-day cumulative drainage | 66/306 | Adjusted OR 1.12 (1.06-1.19) | *z*=3.84 | <0.001 |
| Continuous-exposure analysis | Per 10-percentage-point decrease in relative reduction from D1 to D7 | 66/306 | Adjusted OR 1.51 (1.25-1.82) | *z*=4.30 | <0.001 |

**Note:** OR denotes odds ratio; CI, confidence interval; *z*, Wald *z* statistic; *P*, two-sided *P* value; *n*, sample size; mL, milliliters; and D1 and D7, the first and seventh consecutive 24-h postoperative intervals, respectively. The strict outcome required that the patient first meet the primary day-30 outcome and, within 30 postoperative days, undergo at least two aspirations in total or drain reinsertion because of persistent or recurrent fluid accumulation. The alternative outcome analysis that removed the original-drain-retention component retained all 306 patients and was based on 57 alternative-outcome events. The extended models including pT/ypT category or operating surgeon were based on the 66 primary-outcome events. Except for the complete-case analysis, adjusted models were based on 20 multiply imputed datasets. Posterior-probability-weighted models used patient-level cluster-robust standard errors. All adjusted models included age, body mass index, diabetes mellitus, neoadjuvant systemic therapy, resected specimen weight, operative time, and number of dissected lymph nodes; extended models additionally included pT/ypT category or operating surgeon. pT denotes pathologic T category, and ypT denotes post-neoadjuvant pathologic T category.

**Supplementary Table 2. Exploratory comparison of trajectory classification with simpler drainage measures**

| **Model** | **Events/sample size** | **AIC** | **C statistic (95% CI)** | **Brier score** |
| --- | --- | --- | --- | --- |
| Clinical-covariate model | 61/289 | 286.4 | 0.741 (0.679-0.803) | 0.145 |
| Clinical covariates + D7 drainage | 61/289 | 252.1 | 0.844 (0.795-0.893) | 0.114 |
| Clinical covariates + 7-day cumulative drainage | 61/289 | 257.6 | 0.832 (0.779-0.885) | 0.119 |
| Clinical covariates + relative reduction from D1 to D7 | 61/289 | 266.9 | 0.808 (0.750-0.866) | 0.127 |
| Clinical covariates + trajectory class | 61/289 | 250.8 | 0.849 (0.800-0.898) | 0.111 |
| Clinical covariates + D7 drainage + trajectory class | 61/289 | 251.1 | 0.852 (0.804-0.900) | 0.110 |
| Clinical covariates + 7-day cumulative drainage + trajectory class | 61/289 | 251.0 | 0.853 (0.804-0.902) | 0.110 |
| Clinical covariates + relative reduction from D1 to D7 + trajectory class | 61/289 | 251.6 | 0.851 (0.802-0.900) | 0.111 |

**Note:** *n* denotes sample size; D1 and D7 denote the first and seventh consecutive 24-h postoperative intervals, respectively; AIC, Akaike information criterion; CI, confidence interval; and *P*, two-sided *P* value. The C statistic measures model discrimination, and the Brier score measures the overall error of predicted probabilities. *χ²* is the likelihood-ratio test statistic. Model comparison was restricted to 289 patients with complete covariate data (61 events) and used the same standard multivariable logistic regression framework. Clinical covariates were age, body mass index, diabetes mellitus, neoadjuvant systemic therapy, resected specimen weight, operative time, and number of dissected lymph nodes. The AIC, C statistic, and Brier score are apparent performance estimates in the current 289 complete cases; no optimism correction was performed. The 95% CIs for the C statistic and its differences were estimated using 1,000 patient-level nonparametric bootstrap resamples, with models refitted in each resample. Compared with the corresponding simpler-measure model, adding trajectory class yielded the following results: for the D7-drainage model, likelihood-ratio *χ²*(2)=5.00, *P*=0.082, and an increase in the C statistic of 0.008 (95% CI, −0.004 to 0.020; *P*=0.191); for the 7-day cumulative-drainage model, likelihood-ratio *χ²*(2)=10.60, *P*=0.005, and an increase in the C statistic of 0.021 (95% CI, 0.006–0.036; *P*=0.006); and for the D1-to-D7 relative-reduction model, likelihood-ratio *χ²*(2)=19.30, *P*<0.001, and an increase in the C statistic of 0.043 (95% CI, 0.025–0.061; *P*<0.001). Lower AIC and Brier scores indicate better relative fit or lower overall prediction error.
